# Progressive multifocal leukoencephalopathy and black fungus in a patient with rheumatoid arthritis without severe lymphocytopenia

**DOI:** 10.1099/jmmcr.0.005053

**Published:** 2016-08-30

**Authors:** Marieke J. A. de Regt, Jean-Luc Murk, Tilman Schneider-Hohendorf, Mike P. Wattjes, Andy I. M. Hoepelman, Joop E. Arends

**Affiliations:** ^1^​ Department of Internal Medicine and infectious Diseases, University Medical Centre Utrecht, the Netherlands; ^2^​ Department of Medical Microbiology, University Medical Centre Utrecht, Utrecht, the Netherlands; ^3^​ Department of Neurology, University of Munster, Munster, Germany; ^4^​ Department of Radiology and Nuclear Medicine, VU University Medical Centre, Amsterdam, the Netherlands

**Keywords:** Progressive multifocal leukoencephalopathy (PML), immune reconstitution inflammatory syndrome (IRIS), rheumatoid arthritis, leukoencephalopathy, white matter lesions, paresis, JC-polyomavirus, Maraviroc, Mirtazapine

## Abstract

**Introduction:**

Progressive multifocal leukoencephalopathy (PML) is a rare demyelinating brain infection caused by JC polyomavirus (JCV), primarily seen in patients with severely compromised cellular immunity. Clinical presentation varies depending on the affected white matter. PML prognosis is variable and effective treatments are lacking.

**Case presentation:**

A 75-year-old Chinese woman with type 2 diabetes mellitus, chronic kidney disease and rheumatoid arthritis, treated with low-dose methotrexate and prednisolone for 2.5 years, developed a *Pleurostomophora richardsiae* infection of her left arm. After 6 months of treating this rare black fungus infection with voriconazole, surgery and immunosuppression discontinuation, she presented with progressive afebrile encephalopathy with right-sided hemiparesis. There were no signs of inflammation or metabolic abnormalities. Brain magnetic resonance imaging revealed diffuse frontal white matter lesions and a cerebrospinal fluid PCR confirmed PML due to JC virus. Severe lymphopenia was never present, and at PML diagnosis, CD4 and CD8 T-cell counts were 454 mm^−3^ and 277 mm^−3^. CD8 T-cells were able to respond to JCV VP1 peptide stimulation with TNFα secretion. Peripheral B-cell count was only 8 mm^−3^. Mirtazapine and Maraviroc were started, but unfortunately, she rapidly deteriorated and died 5 weeks after PML diagnosis.

**Conclusion:**

Although peripheral lymphocyte counts were never low and CD4 T-cell count was close to normal, the persistent black fungus infection was a hallmark of severely compromised cellular immunity. The unexpected extremely low absolute B-cell count might suggest a protective role for B-cells. The paradoxical, clinical PML onset months after immunosuppressive discontinuation suggests that it was only discovered in the context of an immune reconstitution inflammatory syndrome.

## Introduction

Progressive multifocal leukoencephalopathy (PML) is a rare demyelinating lytic brain infection caused by JC polyomavirus (JCV) (Hirsch *et al.*, 2013). Over half of the general population becomes infected with JC virus during life, causing a lifelong asymptomatic infection in various organs, including the bone marrow and kidneys (Hirsch *et al.*, 2013). It is only when the cellular immune system becomes severely compromised, mainly due to human immunodeficiency virus (HIV), transplantation or immune modulating monoclonal antibodies (specifically natalizumab), that JCV reactivation causes PML (Hirsch *et al.*, 2013). Specific mutations and genomic rearrangements found in JC DNA derived from PML brain tissue are thought to lead to viral tropism for the brain and increased virulence. At what stage JCV crosses the blood–brain barrier to infect the brain remains unclear.

PML has a subacute onset and symptoms are highly variable, depending on the affected white matter. Typical symptoms are cognitive decline, vision loss, impaired speech and paralysis. Signs of inflammation are generally absent (Hirsch *et al.*, 2013). Asymptomatic PML has been described in multiple sclerosis (MS) patients (Wattjes *et al.*, 2015). In general, prognosis of PML is poor depending on the capability of restoring the host immune system (Hirsch *et al.*, 2013).

In this report, we present a unique case of a patient who developed symptomatic PML months after discontinuation of her rather mild immunosuppressive therapy.

## Case report

A 75-year-old Chinese woman with type 2 diabetes mellitus, chronic kidney disease and rheumatoid arthritis received treatment for a rare *Pleurostomophora richardsiae* infection of her left arm. This ‘black fungus’ was cultured from two separate sterile pus cultures and one wound culture of the left arm and sent to the CBS Fungal Biodiversity Centre and Radboud University Medical Centre for determination. She developed this fungal infection after using methotrexate (7.5 mg weekly) and prednisolone (7.5 mg daily) for 2.5 years. After 6 months of voriconazole, repeated surgical eradication and immunosuppression discontinuation, she presented with fatigue and light-headedness. A week later, she was admitted with progressive weakness, paresis of her right arm and fluctuating apathy. Physical examination demonstrated afebrile encephalopathy with right-sided hemiparesis. A brain magnetic resonance imaging (MRI) was made and a lumbar puncture was performed. Acyclovir was started empirically.

## Investigations

**Fig. 1.**
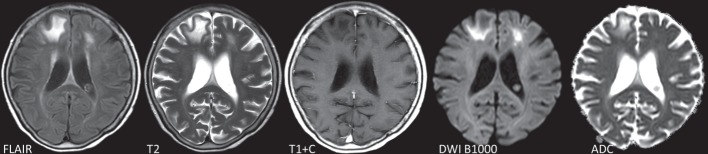
MRI brain showing white matter lesions in both frontal lobes affecting the deep and juxtacortical white matter. The lesions show a spreading along the white matter tract entering the contralateral hemisphere via the genu anterius of the corpus callosum.

**Fig. 2.**
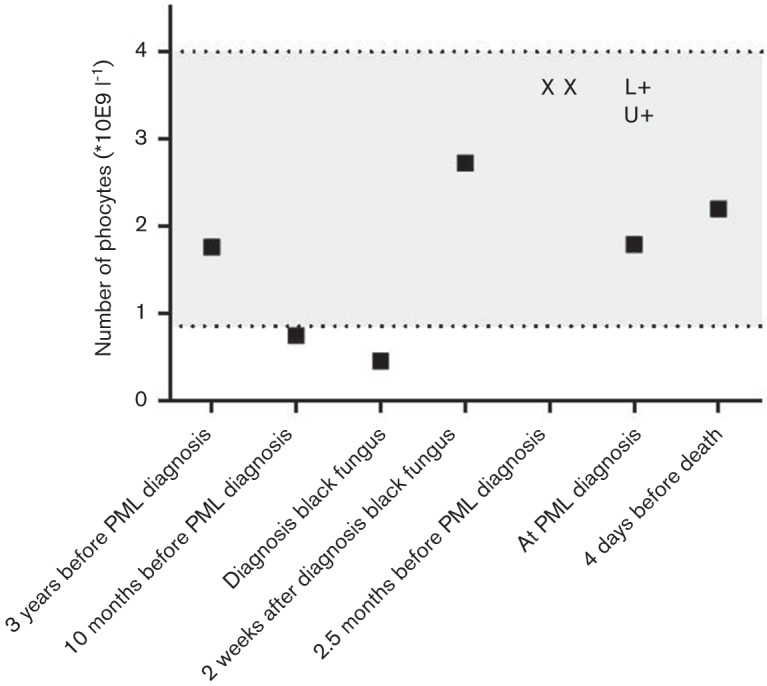
Patient’s lymphocyte counts over the course of time (denoted by squares). Legend extra symbols: X= negative JCV PCR on blood, L+= positive JCV PCR on CSF (2 log2 copies ml^−1^), U+=positive JCV PCR on urine (8 log2 copies ml^−1^).

**Fig. 3.**
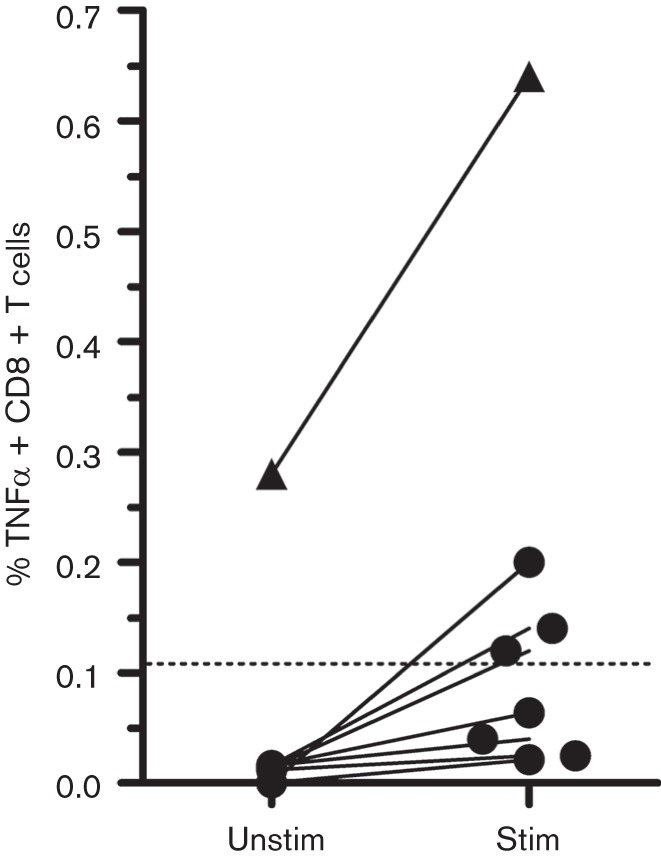
CD8 T-cell TNFα secretion after *in vitro* stimulation with JCV VP1 peptide. Circles indicate seven healthy control subjects; triangles indicate the patient’s samples. The dotted line indicates the detection threshold. Unstim = TNFα secretion of unstimulated CD8 T-cells; stim = TNFα secretion of CD8 T-cells stimulated with JCV VP1 peptide pool for 6 h.

Laboratory results showed normal electrolytes, unaltered renal function (creatinine 181 umol l^−1^), C-reactive protein 7.1 mg l^−1^ and leucocyte count 9.4*10E9 l^−1^, with normal differentiation. There were no signs of hepatic failure. Brain MRI revealed white matter lesions in both frontal lobes affecting the deep and juxtacortical white matter (Fig. 1). The lesions showed spreading along the white matter tract entering the contralateral hemisphere via the genu anterius of the corpus callosum. A lumbar puncture showed no pleiocytosis (1*106 l^−1^ leucocytes with no erythrocytes) and only minimally elevated protein (0.51 g l^−1^) and normal glucose (4.7 mmol l^−1^) levels in the cerebrospinal fluid (CSF). HIV, syphilis, borreliosis and toxoplasmosis were ruled out serologically. CSF tested negative by PCR for human herpes viruses and enteroviruses while positive for JCV establishing the diagnosis of PML. Retrospectively performed PCRs on two consecutive blood samples taken 2 months before PML diagnosis were negative for JCV. A urine sample taken 2 weeks after diagnosis showed shedding of JCV in the urine. Peripheral lymphocyte counts were always normal, except during the period of active and still untreated black fungus infection, when they were marginally depressed to levels between 0.46 and 0.75*109 l^−1^ (Fig. 2). CD4 and CD8 T-cell counts at PML diagnosis were 454 mm^−3^ and 277 mm^−3^, respectively, whereas the absolute peripheral B-cell count was only 8 mm^−3^ (with normal total IgG, IgM and IgA levels). CD8 T-cells, isolated at PML diagnosis, reacted to JCV VP1 peptide stimulation with TNFα secretion (Fig. 3). For this assay, a JCV VP1 peptide pool (PepTivator^®^) was purchased from Miltenyi Biotec and used according to the manufacturer’s instructions. In brief, thawed peripheral blood mononuclear cell (PBMC) was either rested for 2 h and incubated with 1 µg ml^−1^ brefeldin A for additional 4 h or stimulated for 6 h with 20 µl of PepTivator^®^ stock solution for 5×106 PBMC/ml, as well as addition of 1 µg ml^−1^ brefeldin A after 2 h. Harvested cells were then stained with fluorochrome-conjugated antibodies against CD3, CD4, CD8 and CD56; fixated and permeabilized and intracellularly stained with a fluorochrome-conjugated antibody against TNFα (Biolegend) as previously described (Breuer *et al.*, 2014).

## Diagnosis

There was a PML due to JC virus reactivation in rheumatoid arthritis patients with severe immunodeficiency.

## Treatment

Mirtazapine was started to block glial cell infection through the serotonin receptor 5HT2a (Hirsch *et al.*, 2013) and, 4 weeks thereafter (when permission for off-label use was received), Maraviroc, a CCR5 antagonist used in HIV, was initiated (Giacomini *et al.*, 2014).

## Outcome and follow-up

Unfortunately, our patient quickly deteriorated due to PML progression, which led to her death 5 weeks after diagnosis and treatment initiation.

## Discussion

PML is a rare demyelinating lytic brain infection caused by JCV (Hirsch *et al.*, 2013). Over half of the general population becomes infected with JC virus during life, causing a lifelong asymptomatic infection, unless the immune system is severely compromised due to AIDS, transplantation or immune modulating monoclonal antibodies, specifically natalizumab (Hirsch *et al.*, 2013). Asymptomatic PML has been described in MS patients (Wattjes *et al.*, 2015). Only few PML cases have been reported with rheumatoid arthritis (Palazzo & Yahia, 2012). Symptoms have a subacute onset and are highly variable, depending on the affected white matter. Typical symptoms are cognitive decline, vision loss, impaired speech and paralysis. Signs of inflammation are generally absent (Hirsch *et al.*, 2013).

In retrospect, the persistent black fungus infection in our patient was a hallmark of severely compromised cellular immunity. Surprisingly, peripheral lymphocyte counts were never extremely low and both CD4 and CD8 T-cell counts at PML diagnosis were (close to) normal. In addition, CD8 T-cells secreted TNFα after stimulation with a JCV VP1 peptide pool. This strongly indicates the presence of JCV-specific lymphocytes, which can be found in the majority of healthy individuals (Du Pasquier *et al.*, 2004; Koralnik *et al.*, 2001). The only striking result was an exceptionally low absolute B-cell count (8 mm^−3^). Although the role of B-cell depletion in PML pathogenesis is unknown, the increased PML risk in rituximab-treated patients suggests a protective role for B-cells. The paradoxical clinical onset of PML months after immunosuppressives discontinuation and relatively high CD4 count suggests that PML was only discovered in the context of an immune reconstitution inflammatory syndrome (IRIS) (Palazzo & Yahia, 2012), as seen in HIV patients on antiretroviral therapy and in MS patients after natalizumab discontinuation (Palazzo & Yahia, 2012). Only few PML cases have been reported with rheumatoid arthritis (Palazzo & Yahia 2012). Most cases were treated with rituximab and methotrexate and had peripheral lymphopenia. Yet, PML without severe lymphocytopenia has been sparsely reported (Nieuwkamp *et al.*, 2015; Dammeier *et al.*, 2015; Hoepner *et al.*, 2015). Up until now, there has been no specific successful treatment for PML. The most important goal of treatment is to restore immune control over JCV replication by discontinuing immunosuppressives or, in case of HIV, starting antiretroviral therapy. Routine use of corticosteroid therapy in PML IRIS is not recommended, as it may inhibit immune recovery and therefore lead to disease progression (Hirsch *et al.*, 2013). Our patient was treated with Mirtazapine to block glial cell infection via the 5HT2a receptor, which has been used with variable success in several studies (Hirsch *et al.*, 2013). In view of the relatively high levels of (activated) CD4 and CD8 T-cell counts, permission for off-label Maraviroc prescription, a CCR5 antagonist used as antiretroviral agent in HIV-infected patients, was requested (Giacomini *et al.*, 2014). Previously, our group successfully treated three HIV-negative PML patients under the hypothesis that lowering CD4 T-cell activation increases its antiviral function (Middel *et al.*, 2015). Unfortunately, our patient died due to PML progression after only 1 week of Maraviroc therapy (Giacomini *et al.*, 2014).

This case report demonstrates that even patients on relatively mild immunosuppressive drugs and no measured severe lymphopenia may have a compromised cellular immunity and therefore are at risk of developing PML. Moreover, in these patients, PML may develop clinically silent, only becoming symptomatic when immune reconstitution has started.

## References

[R3] Breuer J. Schwab N. Schneider-Hohendorf T. Marziniak M. Mohan H. Bhatia U. Gross C. C. Clausen B. E. Weishaupt C.(2014). Ultraviolet B light attenuates the systemic immune response in central nervous system autoimmunity. Ann Neurol 75 739–758. 10.1002/ana.24165 24771567

[R4] Dammeier N. Schubert V. Hauser T. K. Bornemann A. Bischof F.(2015). Case report of a patient with progressive multifocal leukoencephalopathy under treatment with dimethyl fumarate. BMC Neurol 15 108. 10.1186/s12883-015-0363-8 26152311 PMC4495627

[R10] Du Pasquier R. A. Schmitz J. E. Jean-Jacques J. Zheng Y. Gordon J. Khalili K. Letvin N. L. Koralnik I. J.(2004). Detection of JC virus-specific cytotoxic T lymphocytes in healthy individuals. J Virol 78 10206–10210. 10.1128/JVI.78.18.10206-10210.2004 15331755 PMC514969

[R5] Giacomini P. S. Rozenberg A. Metz I. Araujo D. Arbour N. Bar-Or A. Maraviroc in Multiple Sclerosis–Associated PML–IRIS (MIMSAPI) Group(2014). Maraviroc and JC virus-associated immune reconstitution inflammatory syndrome. N Engl J Med 370 486–488. 10.1056/NEJMc1304828 24476450 PMC5052063

[R6] Hirsch H. H. Kardas P. Kranz D. Leboeuf C.(2013). The human JC polyomavirus (JCPyV): virological background and clinical implications. APMIS 121 685–727. 10.1111/apm.12128 23781977

[R2] Hoepner R. Faissner S. Klasing A. Schneider R. Metz I. Bellenberg B. Lukas C. Altmeyer P. Gold R. Chan A.(2015). Progressive multifocal leukoencephalopathy during fumarate monotherapy of psoriasis. Neurol Neuroimmunol Neuroinflamm 2, e85. 10.1212/NXI.0000000000000085 25798449 PMC4360799

[R1] Koralnik I. J. Du Pasquier R. A. Letvin N. L.(2001). JC virus-specific cytotoxic T lymphocytes in individuals with progressive multifocal leukoencephalopathy. J Virol 75 3483–3487. 10.1128/JVI.75.7.3483-3487.2001 11238876 PMC114143

[R7] Middel A. Arends J. E. van Lelyveld S. F. Otto S. Schuurman R. Frijns C. J. Tesselaar K. Hoepelman A. I.(2015). Clinical and immunologic effects of maraviroc in progressive multifocal leukoencephalopathy. Neurology 85 104–106. 10.1212/WNL.0000000000001713 26041329

[R8] Nieuwkamp D. J. Murk J. L. van Oosten B. W. Cremers C. H. Killestein J. Viveen M. C. Van Hecke W. Frijlink D. W. Wattjes M. P. PML in Dutch MS Patients Consortium(2015). PML in a patient without severe lymphocytopenia receiving dimethyl fumarate. N Engl J Med 372 1474–1476. 10.1056/NEJMc1413724 25853764

[R9] Palazzo E. Yahia S. A.(2012). Progressive multifocal leukoencephalopathy in autoimmune diseases. Joint Bone Spine 79 351–355. 10.1016/j.jbspin.2011.11.002 22281228

[R11] Wattjes M. P. Vennegoor A. Steenwijk M. D. de Vos M. Killestein J. van Oosten B. W. Mostert J. Siepman D. A. Moll W.(2015). MRI pattern in asymptomatic natalizumab- associated PML. J Neurol Neurosurg Psychiatry 86 793–798. 10.1136/jnnp-2014-308630 25205744

